# *N*-Glycoprofiling of SLC35A2-CDG: Patient with a Novel Hemizygous Variant

**DOI:** 10.3390/biomedicines11020580

**Published:** 2023-02-16

**Authors:** Rebeka Kodríková, Zuzana Pakanová, Maroš Krchňák, Mária Šedivá, Sergej Šesták, Filip Květoň, Gábor Beke, Anna Šalingová, Katarína Skalická, Katarína Brennerová, Emília Jančová, Peter Baráth, Ján Mucha, Marek Nemčovič

**Affiliations:** 1 Institute of Chemistry, Centre of Glycomics, Slovak Academy of Sciences, Dúbravská cesta 9, 841 04 Bratislava, Slovakia; 2 Department of Genomics and Biotechnology, Institute of Molecular Biology, Slovak Academy of Sciences, Dúbravská cesta 21, 845 51 Bratislava, Slovakia; 3 National Institute of Children’s Diseases, Center for Inherited Metabolic Disorders, Limbová 1, 833 40 Bratislava, Slovakia; 4 Laboratory of Clinical and Molecular Genetics, National Institute of Children’s Diseases, Limbová 1, 833 40 Bratislava, Slovakia; 5 Department of Paediatrics, Faculty of Medicine of Comenius University and National Institute for Children’s Diseases, Limbová 1, 833 40 Bratislava, Slovakia

**Keywords:** congenital disorders of glycosylation, glycoprofiling, SLC35A2-CDG

## Abstract

Congenital disorders of glycosylation (CDG) are a group of rare inherited metabolic disorders caused by a defect in the process of protein glycosylation. In this work, we present a comprehensive glycoprofile analysis of a male patient with a novel missense variant in the *SLC35A2* gene, coding a galactose transporter that translocates UDP-galactose from the cytosol to the lumen of the endoplasmic reticulum and Golgi apparatus. Isoelectric focusing of serum transferrin, which resulted in a CDG type II pattern, was followed by structural analysis of transferrin and serum *N*-glycans, as well as the analysis of apolipoprotein CIII *O*-glycans by mass spectrometry. An abnormal serum *N*-glycoprofile with significantly increased levels of agalactosylated (Hex3HexNAc4-5 and Hex3HexNAc5Fuc1) and monogalactosylated (Hex4HexNAc4 ± NeuAc1) *N*-glycans was observed. Additionally, whole exome sequencing and Sanger sequencing revealed de novo hemizygous c.461T > C (p.Leu154Pro) mutation in the *SLC35A2* gene. Based on the combination of biochemical, analytical, and genomic approaches, the set of distinctive *N*-glycan biomarkers was characterized. Potentially, the set of identified aberrant *N*-glycans can be specific for other variants causing SLC35A2-CDG and can distinguish this disorder from the other CDGs or other defects in the galactose metabolism.

## 1. Introduction

Congenital disorders of glycosylation (CDG) are a group of >140 rare metabolic disorders, the majority of which are caused by defects in the *N*-linked glycosylation of proteins [1]. Clinical manifestation of CDG is associated with a wide range of symptoms on the level of multiple organ systems, the most affected being the nervous system with the typical presence of hypotonia, developmental delay, and seizures [2,3]. Numerous clinical signs, specific to different types of CDG, make their diagnosis challenging.

SLC35A2-CDG (CDG-IIm, MIM 300896) is a rare disorder caused by a mutation in the *X*-linked *SLC35A2* gene on chromosome Xp11.23. *SLC35A2* encodes the major galactose Golgi-localized transporter, a member of the nucleotide-sugar transporter family that selectively imports UDP-galactose from the cytoplasm to the Golgi, where it serves as a glycosyl donor for composing the complex type *N*-glycans with sialic acid [4,5]. This gene mutation results in hypogalactosylation of *N*-glycans characterized by the presence of truncated β-GlcNAc-terminated-glycans due to the absence of UDP-galactose molecules in the Golgi [6,7]. Approximately 65 cases of SLC35A2-CDG have been reported so far with the majority of missense/nonsense types of mutations and some small deletion mutations (HGMD^®^ Professional). The majority of these cases are pathogenic de novo variants affecting more females than males due to X-chromosome inactivation [8]. In the literature, the clinical manifestation of SLC35A2-CDG includes typical neurological symptoms, such as failure to thrive, abnormal brain structures, developmental delay, and epilepsy, but also other clinical features such as muscular hypotonia, skeletal abnormality, facial dysmorphism, or liver defects. Diverse disease severity and clinical manifestation can be caused by variability in the type of mutation (frameshift vs. missense), the degree of *X*-chromosome inactivation, or the level of mosaicism [5,7,9].

We present molecular and biochemical data of the novel de novo hemizygous missense variant in the *SLC35A2* gene, revealed by whole exome sequencing (WES). A male patient with diabetes mellitus type I, developmental delay, speech disorder, and hepatopathy with elevated AST and ALT underwent selective CDG screening. Based on a positive result of isoelectric focusing of serum transferrin, we comprehensively analyzed the *N*-glycoprofile of the serum transferrin and the patient’s blood serum, and *O*-glycoprofile of apolipoprotein CIII by MALDI-TOF mass spectrometry (MS). Additionally, the novel *SLC35A2* mutation identified by WES was confirmed with Sanger sequencing.

## 2. Materials and Methods

### 2.1. Samples and Participants

A 9-year-old male patient was suffering from repeating periods of diabetic decompensation, delayed psychomotor development, speech disorder, hepatopathy, increased levels of AST, ALT, and short stature. Based on the clinical manifestation, serum and blood samples from the patient and his parents were obtained for further analyses from the National Institute of Children’s Diseases according to standard protocols. Informed consent was given by both parents. As negative controls, sera from healthy individuals, 1 premixed serum pool from 10 healthy volunteers, and commercially available serum from human male AB plasma obtained from Merck KGaA (Darmstadt, Germany) were used. The experimental study protocol was reviewed and approved by the hospital ethics committee (Ethics Committee of National Institute of Children Diseases).

### 2.2. Isoelectric Focusing of Transferrin

Isoelectric focusing (IEF) of serum transferrin (Tf) was performed using a PhastSystem appliance (GE Healthcare, Chicago, IL, USA) as described in the literature [10,11]. After the iron saturation, serum was applied to a PhastGel matrix (pH 5–8) and proteins were separated based on their pI values. Tf isoforms were specifically immunofixed with polyclonal anti-Tf rabbit antibodies (Dako, Glostrup, Denmark) and other serum proteins were washed from the gel. Immunofixed Tf isoforms were stained with Coomassie Brilliant Blue R-350.

### 2.3. Analysis of Serum N-Glycans

Analysis of serum *N*-glycans was performed as described previously. Briefly, serum was denatured by the combination of heat and the addition of SDS. *N*-glycans were subsequently (i) released by PNGase F (Roche Holding AG, Basel, Switzerland), (ii) isolated by Supelclean ENVI-Carb SPE column (Supelco, Bellefonte, PA, USA) using 60% acetonitrile with 0.1% TFA for their total elution, and (iii) permethylated [12]. Mass spectra of permethylated *N*-glycan in their sodiated form [M+Na]^+^ were recorded in reflectron positive ion mode (MS) and LIFT mode (MS/MS) using a UltrafleXtreme II MALDI-TOF mass spectrometer (BrukerDaltonics, Billerica, MA, USA). Obtained data were analyzed by FlexAnalysis v.3.4 (BrukerDaltonics, Billerica, MA, USA), ProteinScape v.3.0 (BrukerDaltonics, Billerica, MA, USA), and GlycoWork Bench [13] software programs. All glycan structures presented in this manuscript were confirmed by MS/MS.

### 2.4. Analysis of Transferrin N-Glycans

Tf was isolated from serum using immunoaffinity chromatography. The column was prepared by binding polyclonal rabbit anti-Tf antibodies (DAKO, Glostrup, Denmark) onto the HiTrap NHS medium (GE Healthcare, Chicago, IL, USA) after their desalting by PD-10 desalting column (GE Healthcare, Chicago, IL, USA) according to the manufacturer’s instructions. After washing the unbound serum proteins from the column, Tf was eluted with 0.1 M glycine-HCl (pH 2.7), and the eluate was immediately neutralized by 1 M Tris (pH 8.5) to reclaim the neutral pH of the sample. *N*-glycans from isolated Tf were released and analyzed as described above (see analysis of serum *N*-glycans).

### 2.5. Analysis of Apolipoprotein C-III

Analysis of apolipoprotein C-III, the marker of O-glycosylation defects, was performed as described previously [14]. Briefly, the apolipoprotein C-III-enriched fraction of serum proteins was obtained from whole serum using C8 SPE (Supelclean LC-8, 100 mg, SupelCo, Bellefonte, PA, USA). After column washing, apolipoprotein C-III was eluted with 70% acetonitrile with the addition of 0.1% TFA and analyzed in linear positive ion mode using an UltrafleXtreme II MALDI-TOF mass spectrometer (BrukerDaltonics, Billerica, MA, USA). Obtained data were analyzed using a FlexAnalysis v.3.4 software program (BrukerDaltonics, Billerica, MA, USA).

### 2.6. DNA Isolation, Whole Exome Sequencing (WES), and Data Analysis

DNA was isolated from the blood of the patient and parents using a Quick-DNA Miniprep Kit (Zymo Research, Irvine, CA, USA). Whole exome sequencing was performed at the Institute of Applied Biotechnologies (Olomouc, Czech Republic). WES with a 150-bp paired-end read length for DNA samples was performed by next-generation sequencing (NGS) using the NovaSeq 6000 platform (Illumina, San Diego, CA, USA) and a Twist Human Core Exome library kit (Twist Biosciences, South San Francisco, CA, USA). Confirmation analysis was performed using Sanger sequencing.

Quality control of WES data was achieved with FastQC v0.72 [15]. After the quality control, the reads were trimmed by Trimmomatic v0.38.1 [16] and mapped to the human genome build GRCh38 using BWA-MEM v0.7.17.1 [17,18]. The variants were identified following the GATK (Genome Analysis Toolkit) v4.1.9.0 [19] best practices for germline short variant discovery (SNPs + Indels). The identified variant was verified by NextGene and Geneticist Assistant software (Softgenetics, State College, PA, USA). The variants were annotated with the SNPSift Annotate v4.3 [20] tool using dbSNP build 138 [21], ClinVar (release date: 15 June 2020) [22], and Varsome databases.

### 2.7. Sanger Sequencing

The single nucleotide variant identified by exome sequencing was confirmed using Sanger sequencing of the genomic DNA isolated from the patient’s and his parents’ blood. The primer pairs used for the amplification of the region of genomic DNA around the mutation were designed. The forward primer used was 5′-CCCAGTCACACAGCCAGGAA-3′ (298 bp upstream of the mutation) and the reverse primer was 5′- CGGGTGGCCACGGCGGTACC-3′ (321 bp downstream of the mutation). DNA amplification was performed using GoTaq DNA Polymerase (Promega, Madison, WI, USA). The following conditions were used for amplification: 1 cycle of 95 °C for 2 min, followed by 39 cycles of 95 °C for 30 s, 60 °C for 30 s, 72 °C for 40 s, and a final extension at 72 °C for 5 min. Amplified PCR products were sequenced using the PCR primers as sequencing primers.

### 2.8. Bioinformatics

ClustalW 2.1 [23] (available at https://www.genome.jp/tools-bin/clustalw, accessed on 8 October 2022) was used for multiple sequence alignment (MSA). For predicting pathogenicity in silico, we used combined annotation dependent depletion (CADD) [24]. P78381 (SLC35A2) structural models were investigated by PyMol (The PyMOL Molecular Graphics System v1.2r3pre, Schrödinger, LLC., New York, NY, USA), where selected p.Leu154Pro mutagenesis was performed and the selected rotamer was analyzed using the ‘Protein interfaces, surfaces and assemblies’ service PISA at the European Bioinformatics Institute (PDBePISA) [25,26].

### 2.9. Statistics

For the MS analysis, 3 technical replicates from the serum of the patient and 5 individual negative controls (biological replicates) were used. The presented data were calculated from the relative intensities of the sum of total identified *N*-glycans. Relative intensities of single *N*-glycan structures were calculated from the average values of the replicates. Relative standard deviations were calculated. A one-tailed paired *t*-test (α = 0.05) was applied to evaluate the changes in the levels of aberrant hypogalactosylated *N*-glycans.

## 3. Results

### 3.1. Analysis of Transferrin Glycosylation by Isoelectric Focusing and Mass Spectrometry

**Figure 1:**
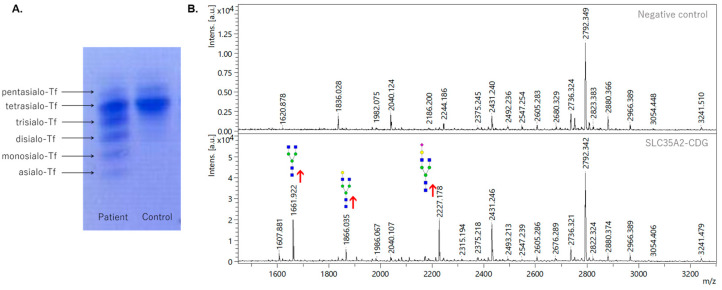
Analysis of glycosylation pattern of serum transferrin from control and SLC35A2-CDG patient samples. (**A**) Isoelectric focusing of serum transferrin. (**B**) MALDI-TOF analysis of permethylated *N*-glycans in sodiated form released from serum transferrin. Red arrows indicate increased intensities of respective *N*-glycan structures in the patient’s sample. Green circle—mannose, blue square—*N*-acetylglucosamine, red triangle—fucose, yellow circle—galactose, purple diamond—sialic acid.

IEF-Tf, as the first approach, revealed an abnormal pattern typical for CDG type II, in which the relative amount of trisialo-Tf is significantly increased. Likewise, the other undersialylated isoforms of transferrin (asialo-Tf, monosialo-Tf, disialo-Tf) showed increased levels in comparison with the control sample (Figure 1A). A simultaneous reduction in the tetrasialo-Tf level was also observed. Further comprehensive *N*-glycoprofile analysis of Tf confirmed the abnormal pattern from IEF-Tf, where significant hypogalactosylation, represented by the increased relative abundance of Hex3HexNAc4 at *m*/*z* 1661.9, Hex4HexNAc4 at *m*/*z* 1865.1, Hex4HexNAc4NeuAc1 at *m*/*z* 2227.2, and Hex5HexNAc4NeuAc1 *N*-glycan at *m*/*z* 2431.2 was detected (Figure 1B).

### 3.2. Analysis of Serum Protein Glycosylation by Mass Spectrometry

**Figure 2:**
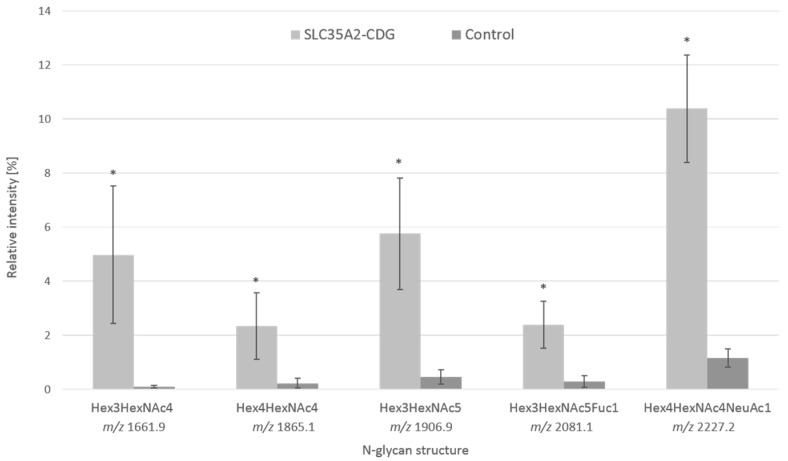
Relative abundance of identified aberrant agalactosylated and monogalactosylated *N*-glycans. Percentage values were calculated from the total amount of *N*-glycans identified in the three technical replicates of the patient’s serum and five individual control sera. Fuc—fucose, Hex—hexose, HexNAc—*N*-acetylhexosamine, NeuAc—sialic acid. * = *p* ≤ 0.05.

To define the patient’s total *N*-glycoprofile, an analysis of total serum *N*-glycans by MALDI-TOF/TOF was performed for the identification of more complex *N*-glycan moieties. The comprehensive analysis revealed an abnormal increase in hypogalactosylated structures. Similar to the Tf *N*-glycoprofile, the signals at *m*/*z* 1661.9, *m*/*z* 1865.1, and *m*/*z* 2227.2 and were present in the spectra in increased relative intensities, but signals at *m*/*z* 1906.9 (Hex3HexNAc5) and *m*/*z* 2081.1 (Hex3HexNAc5Fuc1) were identified at an increased level in the patient’s serum *N*-glycoprofile (Figure S1). Overall, the relative intensities of agalactosylated and monogalactosylated structures were increased in the patient’s sample. The major aberrations observed in serum were in the levels of two agalactosylated glycans (Hex3HexNAc4 and Hex3HexNAc5) with a 50.0- and 12.3-fold increase. The level of another agalactosylated glycan Hex3HexNAc5Fuc1 was 8.1-fold higher than in the control sample. In the case of monogalactosylated glycans, the relative level of Hex4HexNAc4 was 9.8-fold higher, while the level of its sialylated form Hex4HexNAc4NeuAc1 was 8.9-fold higher than in the negative control. The differences between the patient and negative controls are shown in Figure 2. A significant increase in the levels of hypogalactosylated *N*-glycans was detected in the patient’s serum sample. Furthermore, decreased relative intensities of some sialylated glycans, such as the most abundant disialo-biantennary Hex5HexNAc4NeuAc2 (2.0-fold decrease), were observed in the patient’s sample. Additionally, whole venous blood *N*-glycoprofile analysis without any prior separation of serum was performed. The same abnormal pattern with significantly increased levels of agalactosylated and monogalactosylated *N*-glycans, as in the patient’s serum, was observed (data not shown). To identify the potential *O*-glycosylation defects, apolipoprotein C-III analysis by MALDI-TOF MS was performed; however, its *O*-glycoprofile did not reveal any significant aberrations (data not shown).

### 3.3. Analysis of DNA and Bioinformatic Processing

**Figure 3:**
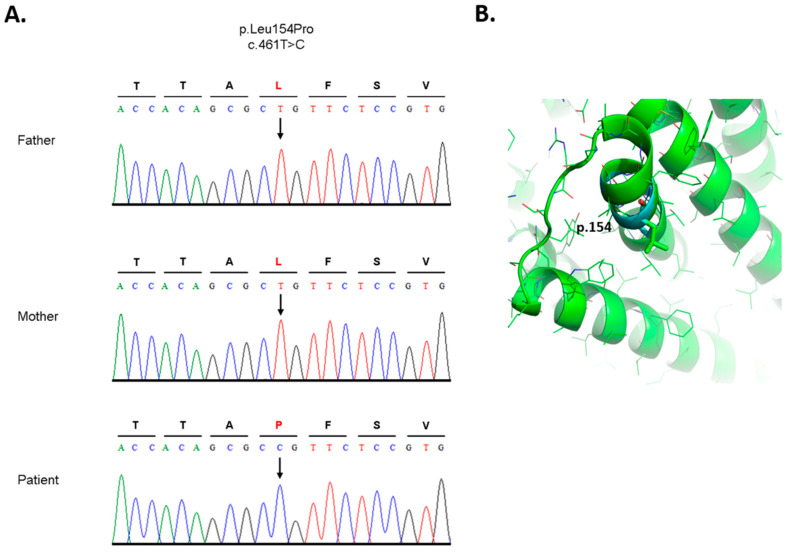
(**A**) Sanger sequencing analysis of the SLC35A2-CDG patient and his parents. Representative sequence chromatograms of unaffected parents and the patient are shown. The protein and cDNA annotations of the identified new variant are shown above. The arrow shows the position of nucleotide exchange of a new variant. (**B**) Tertiary SLC35A2 protein structure. P.154 amino acid selected for in silico mutagenesis p.Leu154Pro and indicated in different colors (atoms are color-coded), leucine—green, superimposed proline—cyan.

Whole exome sequencing of blood gDNA revealed a novel (*de novo*) variant in the *SLC35A2* gene in chromosomal position X:448905448 in reference transcript NM_005660.3. This represented a single nucleotide substitution of thymine for cytosine in the DNA for the codon for amino acid residue 154, changing leucine to proline (HGVS variant description, *SLC35A2* (NM_005660.3): c.461T > C, p.Leu154Pro), confirmed by Sanger sequencing in the patient’s blood gDNA. This variant was absent in both parents (Figure 3A).

Full conservation of leucine 154 was shown by multiple sequence alignment (MSA) of 30 orthologous vertebrate and invertebrate protein species collected from Uniprot (The Uniprot Consortium) [27]. Three valine substitutions (very similar amino acids) were detected. Moreover, the position 154 was also confirmed by the protein SLC35A2 paralogue annotation. As shown in Figure S2, leucine 154 is conserved within all five SLC35A1-5 proteins at its homologous position. Protein (Acc. No. P78381) structure prediction by Alphafold determined the L154 amino acid in one of the eight alpha-helices [25,26]. Substituted proline (P154), because of its irregular geometry where R-group bonds back to the amino group, causes steric hindrance and destabilizes alpha-helices [28,29]. To provide molecular insights, we induced in silico mutation in the predicted SLC35A2 protein structure utilizing the PyMol software program (p.Leu154Pro) (Figure 3B). Further interfacing residue calculation using PDBePisa indicated a decrease of more than half of the solvent-accessible surface area (BSA) of the p.154Pro (11.54 Å2) compared with p.154Leu (26.84 Å2), and a decline of almost four times the solvation energy (ΔiG) of p.154Pro (0.18 kcal/M) compared with p.154Leu (0.43 kcal/M). The protein functional concussion of position 154 is indicated by in silico analysis using the considering combined annotation dependent depletion (CADD) score. The present variant, p.Leu154Pro, has a CADD score of 27.5 which is in the scope of other pathogenic variants with associated phenotypes [4].

## 4. Discussion

In this study, we report the identification and characterization of a patient with a defect in galactosylation of *N*-glycan antennas: SLC35A2-CDG. His clinical features, such as delayed psychomotor development, hepatopathy, and short stature, were comparatively milder than in previously published cases; however, varying degrees of physical and neurodevelopmental deficiencies are typical features of CDG diversity. Furthermore, a strong gender bias in SL35A2-CDG is observed, as only a few patients described up to date are males [4]. Based on the clinical summary, >80% of the patients exhibit neurological symptoms, developmental delay, intellectual disability, facial dysmorphisms, epilepsy, and skeletal and brain abnormalities [9]. However, a distantly similar male case of SLC35A2-CDG without epilepsy, with only minor neurological involvement and with growth deficiency, has been described recently [4]. A total of 77% of SLC35A2-CDG-affected individuals presented in the study by Ng et al., 2019, displayed clear features of failure to thrive. Liver involvement (such as mildly elevated transaminases) was described in 40% of the patients [9].

The IEF Tf profile of the presented patient revealed a pattern typical for CDG type II [2], with highly elevated levels of trisialo Tf and other hyposialylated isoforms. Interestingly, the IEF screening test for glycoisoforms of transferrin of other previously published SLC35A2-CDG patients [4,9] revealed a normal pattern in the majority of the female confirmed cases. However, in the case of male patients, seven out of eight individuals manifested abnormal patterns [7], similar to the results of the presented male patient. Abnormal distribution of transferrin glycoisoforms of the presented patient was further confirmed by the structural analysis of *N*-glycoprofile of isolated Tf. This result correlates with Tf *N*-glycoprofiles of other SLC35A2-CDG patients [30,31], where truncated *N*-glycans lacking galactose or sialic acid residues were detected.

To exclude diabetes mellitus (DM) as a secondary cause of aberrant Tf glycoprofile, additional IEF Tf was performed on samples from eight other hyperglycemic DM patients; however, the Tf profiles of all of them were within the reference intervals for negative controls (data not shown). The abnormal *N*-glycoprofile, with significant changes in the abundance of hypogalactosylated *N*-glycans, is caused by insufficient transport of UDP-galactose from the cytoplasm to the Golgi, where it serves as a glycosyl donor for the synthesis of the complex glycans. The presence of galactose-deficient glycans, identified by MS, has already been described in Witters et al., 2020, where they studied galactose supplementation in SLC35A2-CDG patients. Their work described the same glycoprofile pattern which lacked galactose and had increased levels of hypogalactosylated glycans—Hex3HexNAc4, Hex4HexAc4, and Hex4HexNAc4NeuAc1 [7]. In the serum sample of the presented patient, we identified additional abnormal agalactosylated *N*-glycans (Hex3HexNAc5 and Hex3HexNAc5Fuc1) that can be included in the panel of serum glycobiomarkers for SLC35A2-CDG.

The presence of aberrant levels of unique *N*-glycans can distinguish SLC35A2-CDG from galactosemic patients and other CDGs with a disrupted pathway in the *N*-glycan processing, such as B4GALT1-CDG, TMEM165-CDG, and defects in the COG subunits. SLC35A2-CDG can be distinguished from galactosemia based on the presence of Hex3HexNAc5, Hex3HexNAc5Fuc1, and Hex4HexNAc4NeuAc1 that were not detected in the sera of galactosemic patients [32]. A similar *N*-glycoprofile was detected in B4GALT1-CDG with the presence of increased levels of truncated glycans with terminal *N*-acetylhexosamines (Hex3HexNAc4, Hex3HexNAc5, Hex3HexNAc5Fuc1) and bi-antennary glycans missing both galactose and sialic acid moieties (Hex4HexNAc4NeuAc1). The difference between B4GALT1-CDG and SLC35A2-CDG lies mainly in the elevated level of Hex3HexNAc4Fuc1 glycan in B4GALT1-CDG [33,34], which was not observed in increased relative intensity in either presented or previously identified SLC35A2-CDG patients. An increase in several complex truncated glycans lacking galactose and sialic acid was reported in TMEM165-CDG [35,36,37]; however, neither the serum nor Tf *N*-glycoprofile of SLC35A2-CDG patients reveals increased levels of monosialylated biantennary glycans with two galactoses (Hex5HexNAc4NeuAc1 ± Fuc1) which were identified in TMEM165-CDG. Increased levels of hypogalactosylated and hyposialylated *N*-glycans were described in the samples from patients with Golgi trafficking defects (COG subunits). These defects are of combined *N* + *O*-glycosylation. Moreover, one of the most significant biomarkers of SLC35A2-CDG, the Hex3HexNAc4 *N*-glycan, was not identified in increased levels in COG-CDG patients. A list of other diseases that could affect galactose metabolism and lead to a similar glycoprofile as SLC35A2-CDG, with respective *N*-glycan biomarkers, is shown in Supplementary Table S1. To identify the potential *O*-glycosylation defects, apolipoprotein C-III analysis by MALDI-TOF MS was performed and in agreement with other previously published SLC35A2-CDG cases [4,6], the *O*-glycoprofile of apolipoprotein C-III was within the range of healthy controls.

Furthermore, we inspected possible mutations in the other genes (*PGM1*, *B4GALT1*, and *TMEM165*), which can cause a similar *N*-glycoprofile as in the presented patient to exclude this scenario. Most of them were intronic SNPs, which were already described in variant databases, e.g., dbSNP [21] and/or ClinVar [22]. All of those described in ClinVar have clinical significance: benign/likely benign. We also found two missense mutations (with clinical significance: benign/likely benign) in *PGM1*:

- mutation at position chr1: 63648630 T > C (dbSNP ID: rs11208257, ClinVar ID: 297885) is homozygous in the patient and one of the parents, the other parent is heterozygous (Supplementary Figure S3),
- mutation chr1:63631761 C > T (dbSNP ID: rs1126728; ClinVar ID: 297874) is heterozygous in the patient and one of the parents, and homozygous in the other parent (Supplementary Figure S4) [38].

Whole exome sequencing showed a de novo (absent in both parents) mutation in SLC35A2 (c.461T > C; p.Leu154Pro). Results from the analysis of the DNA sequence of a selected region of the *SLC35A2* gene of the proband, mother, and father are shown in Supplementary Figures S5–S7. This variant was also confirmed using Sanger sequencing. MSA analysis of multiple SLC35A2 orthologs and paralogs showed that Leu154 is a highly conserved residue. The PDBePisa calculation of the mutated p.Leu154Pro predicted SLC35A2 structure supports the assumption that proline amino acids cause decreased accessibility of the UDP-Gal, leading to abnormal *N*-glycans. Based on in silico analysis using CADD, the presented de novo variant is considered a deleterious substitution. In previously described cases of missense and small deletion mutations in *SLC35A2*, CADD scores revealed values considered deleterious variants [9]. A combination of bioinformatic processing and analysis such as CADD, multiple orthologous and paralogous sequence alignments, and proline characteristics of the de novo Leu154Pro mutation point to a consequential effect on the structure of the UDP-galactose translocator that may affect its function and activity.

The results obtained by the presented analytical approaches revealed a set of abnormal *N*-glycans with the possibility of becoming clinical glycobiomarkers or drug targets for the treatments. The levels of identified potential glycobiomarkers can be monitored during the disease’s progression or improvement during nutritional therapy, presently based on an oral galactose supplementation that can enhance enzyme activity by providing an extra substrate, and so improve galactosylation and the patient’s overall health state [7,39]. Monitoring of the levels of *N*-glycan antennary galactosylation can become an essential tool to observe the process of potential incorporation of externally supplemented galactose during treatment.

## 5. Conclusions

We presented a SLC35A2-CDG male pediatric patient with a relatively mild phenotype. Based on positive results from selective screening (IEF Tf), mass spectrometry analysis of *N*-glycosylation of both transferrin and whole serum was performed and revealed an abnormal *N*-glycoprofile with the presence of hypogalactosylated *N*-glycans, which can serve as new glycobiomarkers for this type of CDG. WES confirmed a de novo hemizygous missense variant p.Leu154Pro, and bioinformatic analysis confirmed its likely pathogenic outcome. A combination of clinical, biochemical, analytical, and genomic approaches is an essential tool in challenging CDG diagnostics.

## Data Availability

Not applicable.

## Supplementary Materials

The following supporting information can be downloaded at: https://www.mdpi.com/article/10.3390/biomedicines11020580/s1, Figure S1. MALDI-TOF/TOF MS of increased hypogalactosylated derivatized *N*-glycans released from the patient’s and control blood serum; Figure S2. Multiple sequence alignment (MSA) results—aligned human protein (P78381) with orthologues; Figure S3. Screenshot from integrative genomics viewer of the mutation at position chr1: 63648630 in gene PGM1; Figure S4. Screenshot from integrative genomics viewer of the mutation at position chr1: 63631761 in gene PGM1; Figure S5. Analysis of DNA sequence of the selected region of SLC35A2 gene—proband; Figure S6. Analysis of DNA sequence of the selected region of SLC35A2 gene—mother; Figure S7. Analysis of DNA sequence of the selected region of SLC35A2 gene—father. Table S1. List of other diseases that could affect galactose metabolism and lead to similar serum glycoprofile as in SLC35A2-CDG, with respective increased levels (+) or normal levels (−) of *N*-glycan biomarkers.

## References

[1] Péanne R., de Lonlay P., Foulquier F., Kornak U., Lefeber D.J., Morava E., Pérez B., Seta N., Thiel C., Van Schaftingen E. (2018). Congenital disorders of glycosylation (CDG): Quo vadis?. Eur. J. Med. Genet..

[2] Chang I.J., He M., Lam C.T. (2018). Congenital disorders of glycosylation. Ann. Transl. Med..

[3] Paprocka J., Jezela-Stanek A., Tylki-Szymańska A., Grunewald S. (2021). Congenital Disorders of Glycosylation from a Neu-rological Perspective. Brain Sci..

[4] Quelhas D., Correia J., Jaeken J., Azevedo L., Lopes-Marques M., Bandeira A., Keldermans L., Matthijs G., Sturiale L., Martins E. (2021). SLC35A2-CDG: Novel variant and review. Mol. Genet. Metab. Rep..

[5] Kodera H., Nakamura K., Osaka H., Maegaki Y., Haginoya K., Mizumoto S., Kato M., Okamoto N., Iai M., Kondo Y. (2013). De Novo Mutations in *SLC35A2* Encoding a UDP-Galactose Transporter Cause Early-Onset Epileptic Encephalopathy. Hum. Mutat..

[6] Vals M.-A., Ashikov A., Ilves P., Loorits D., Zeng Q., Barone R., Huijben K., Sykut-Cegielska J., Diogo L., Elias A.F. (2019). Clinical, neuroradiological, and biochemical features of SLC35A2-CDG patients. J. Inherit. Metab. Dis..

[7] Witters P., Tahata S., Barone R., Õunap K., Salvarinova R., Grønborg S., Hoganson G., Scaglia F., Lewis A.M., Mori M. (2020). Clinical and biochemical improvement with galactose supplementation in SLC35A2-CDG. Genet. Med..

[8] Westenfield K., Sarafoglou K., Speltz L.C., Pierpont E.I., Steyermark J., Nascene D., Bower M., Pierpont M.E. (2018). Mo-saicism of the UDP-Galactose transporter SLC35A2 in a female causing a congenital disorder of glycosylation: A case report. BMC Med. Genet..

[9] Ng B.G., Sosicka P., Agadi S., Almannai M., Bacino C.A., Barone R., Botto L.D., Burton J.E., Carlston C., Chung B.H. (2019). SLC35A2-CDG: Functional characterization, expanded molecular, clinical, and biochemical phenotypes of 30 unreported Individuals. Hum. Mutat..

[10] de Jong G., van Eijk H.G. (1988). Microheterogeneity of human serum transferrin: A biological phenomenon studied by isoelectric focusing in immobilized pH gradients. Electrophoresis.

[11] Hackler R., Arndt T., Kleine T.O., Gressner A.M. (1995). Effect of Separation Conditions on Automated Isoelectric Focusing of Carbohydrate-Deficient Transferrin and Other Human Isotransferrins Using the PhastSystem. Anal. Biochem..

[12] Ziburová J., Nemčovič M., Šesták S., Bellová J., Pakanová Z., Siváková B., Šalingová A., Šebová C., Ostrožlíková M., Lekka D.E. (2021). A novel homozygous mutation in the human ALG12 gene results in an aberrant profile of oligomannose N-Glycans in patient’s serum. Am. J. Med. Genet. A.

[13] Ceroni A., Maass K., Geyer H., Geyer R., Dell A., Haslam S.M. (2008). GlycoWorkbench: A Tool for the Computer-Assisted Annotation of Mass Spectra of Glycans. J. Proteome Res..

[14] Ondruskova N., Honzik T., Kolarova H., Pakanova Z., Mucha J., Zeman J., Hansikova H. (2018). Aberrant apolipoprotein C-III glycosylation in glycogen storage disease type III and IX. Metabolism.

[15] Andrews S. (2010). FastQC: A Quality Control Tool for High throughput Sequence Data. http://www.bioinformatics.babraham.ac.uk/projects/fastqc.

[16] Bolger A.M., Lohse M., Usadel B. (2014). Trimmomatic: A flexible trimmer for Illumina sequence data. Bioinformatics.

[17] Li H., Durbin R. (2009). Fast and accurate short read alignment with Burrows—Wheeler transform. Bioinformatics.

[18] Schneider V.A., Graves-Lindsay T., Howe K., Bouk N., Chen H.-C., Kitts P.A., Murphy T.D., Pruitt K.D., Thibaud-Nissen F., Albracht D. (2017). Evaluation of GRCh38 and de novo haploid genome assemblies demonstrates the enduring quality of the reference assembly. Genome Res..

[19] Poplin R., Ruano-Rubio V., DePristo M.A., Fennell T.J., Carneiro M.O., Van der Auwera G.A., Kling D.E., Gauthier L.D., Levy-Moonshine A., Roazen D. (2017). Scaling accurate genetic variant discovery to tens of thousands of samples. bioRxiv.

[20] Cingolani P., Patel V.M., Coon M., Nguyen T., Land S.J., Ruden D.M., Lu X. (2012). Using Drosophila melanogaster as a Model for Genotoxic Chemical Mutational Studies with a New Program, SnpSift. Front. Genet..

[21] Sherry S.T., Ward M.-H., Kholodov M., Baker J., Phan L., Smigielski E.M., Sirotkin K. (2001). dbSNP: The NCBI database of genetic variation. Nucleic Acids Res..

[22] Landrum M.J., Lee J.M., Benson M., Brown G.R., Chao C., Chitipiralla S., Gu B., Hart J., Hoffman D., Jang W. (2018). ClinVar: Improving access to variant interpretations and supporting evidence. Nucleic Acids Res..

[23] Thompson J.D., Higgins D.G., Gibson T.J. (1994). CLUSTAL W: Improving the sensitivity of progressive multiple sequence alignment through sequence weighting, position-specific gap penalties and weight matrix choice. Nucleic Acids Res..

[24] Rentzsch P., Witten D., Cooper G.M., Shendure J., Kircher M. (2019). CADD: Predicting the deleteriousness of variants throughout the human genome. Nucleic Acids Res..

[25] Jumper J., Evans R., Pritzel A., Green T., Figurnov M., Ronneberger O., Tunyasuvunakool K., Bates R., Žídek A., Potapenko A. (2021). Highly accurate protein structure prediction with AlphaFold. Nature.

[26] Krissinel E., Henrick K. (2007). Inference of Macromolecular Assemblies from Crystalline State. J. Mol. Biol..

[27] The UniProt Consortium (2019). UniProt: The universal protein knowledgebase. Nucleic Acids Res..

[28] Varadi M., Anyango S., Deshpande M., Nair S., Natassia C., Yordanova G., Yuan D., Stroe O., Wood G., Laydon A. (2022). AlphaFold Protein Structure Database: Massively expanding the structural coverage of protein-sequence space with high-accuracy models. Nucleic Acids Res..

[29] Li S.C., Goto N.K., A Williams K., Deber C.M. (1996). Alpha-helical, but not beta-sheet, propensity of proline is determined by peptide environment. Proc. Natl. Acad. Sci. USA.

[30] Van Scherpenzeel M., Steenbergen G., Morava E., Wevers R.A., Lefeber D. (2015). J High resolution mass spectrometry gly-coprofiling of intact transferrin for diagnosis and subtype identification in the congenital disorders of glycosylation. Transl. Res..

[31] Dörre K., Olczak M., Wada Y., Sosicka P., Grüneberg M., Reunert J., Kurlemann G., Fiedler B., Biskup S., Hörtnagel K. (2015). A new case of UDP-galactose transporter deficiency (SLC35A2-CDG): Molecular basis, clinical phenotype, and therapeutic approach. J. Inherit. Metab. Dis..

[32] Liu Y., Xia B., Gleason T.J., Castañeda U., He M., Berry G.T., Fridovich-Keil J.L. (2012). N- and O-linked glycosylation of total plasma glycoproteins in galactosemia. Mol. Genet. Metab..

[33] Guillard M., Morava E., van Delft F.L., Hague R., Körner C., Adamowicz M., Wevers R.A., Lefeber D.J. (2011). Plasma N-Glycan Profiling by Mass Spectrometry for Congenital Disorders of Glycosylation Type II. Clin. Chem..

[34] Staretz-Chacham O., Noyman I., Wormser O., Abu Quider A., Hazan G., Morag I., Hadar N., Raymond K., Birk O.S., Ferreira C.R. (2020). B4GALT1-congenital disorders of glycosylation: Expansion of the phenotypic and molecular spectrum and review of the literature. Clin. Genet..

[35] Nilsson I., Sääf A., Whitley P., Gafvelin G., Waller C., von Heijne G. (1998). Proline-induced disruption of a transmembrane α-helix in its natural environment. J. Mol. Biol..

[36] Foulquier F., Amyere M., Jaeken J., Zeevaert R., Schollen E., Race V., Bammens R., Morelle W., Rosnoblet C., Legrand D. (2012). TMEM165 Deficiency Causes a Congenital Disorder of Glycosylation. Am. J. Hum. Genet..

[37] Xia B., Zhang W., Li X., Jiang R., Harper T., Liu R., Cummings R.D., He M. (2013). Serum N-glycan and O-glycan analysis by mass spectrometry for diagnosis of congenital disorders of glycosylation. Anal. Biochem..

[38] Robinson J.T., Thorvaldsdóttir H., Winckler W., Guttman M., Lander E.S., Getz G., Mesirov J.P. (2011). Integrative genomics viewer. Nat. Biotechnol..

[39] Abuduxikuer K., Wang J.-S. (2021). Four New Cases of SLC35A2-CDG With Novel Mutations and Clinical Features. Front. Genet..

