# Supplementary material for: N-Glycoprofiling of SLC35A2-CDG: Patient with a Novel Hemizygous Variant
Source: Biomedicines. 2023 Feb 16;11(2):580. doi: 10.3390/biomedicines11020580 (PMC9952902; doi:10.3390/biomedicines11020580)
Supplement: Supplementary file 1 [file biomedicines-11-00580-s001.zip › biomedicines-2134710-supplementary.pdf]

Supplementary material

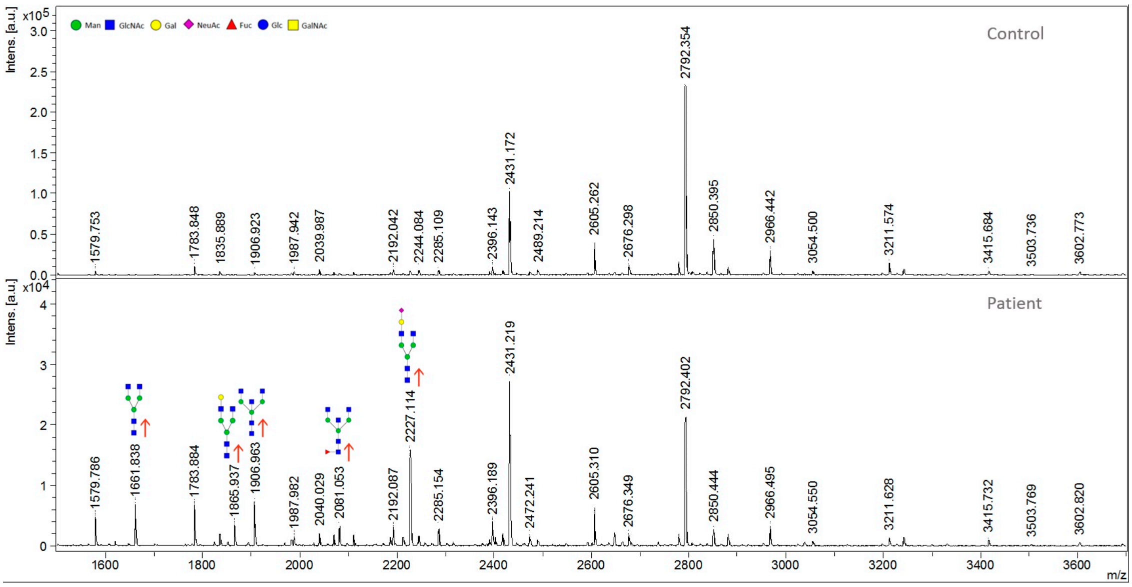

Figure S1. MALDI-TOF/TOF MS of increased hypogalactosylated derivatized N-glycans released from the patient's and control blood serum.

Organism name	Uniprot ID		
H. sapiens	P78381	KILTTALFSVLMLNRSL	
P. abelii	A0A2J8R8J3	KILTTALFSVLMLNRSL	
C. syrichta	A0A1U7UF56	KILTTALFSVLMLNRSL	
P. macrocephalus	A0A2Y9FGB5	KILTTALFSVLMLNRSL	
B. taurus	Q6YC49	KILTTALFSVSMLSKKL	
E. caballus	F7C7T4	KILTTALFSVFMLNRSL	
C. lupus familiaris	O77592	KILTTALFSVSMLSKKL	
V. vulpes	A0A3Q7SA97	KILTTALFSVLMLNRSL	
P. pardus	A0A6P4T3G7	KILTTALFSVLMLNRSL	
O. rosmarus divergens	A0A2U3VNZ3	KILTTALFSVLMLNRSL	Mammals
P. vampyrus	A0A6P3RXC5	KILTTALFSVLMLNRSL	
P. discolor	A0A6J2MGD1	KILTTALFSVLMLNRSL	
M. musculus	Q9R0M8	KILTTALFSVLMLNRSL	
C. griseus	Q99PG7	KILTTALFSVLMLNRSL	
M. auratus	A0A1U8CKB9	KILTTALFSVLMLNRSL	
D. ordii	A0A1S3GII7	KILTTALFSVLMLNRSL	
H. glaber	A0A0P6J5Z9	KILTTALFSVLMLNRSL	
E. europaeus	A0A1S3A515	KILTTALFSVLMLNRSL	
L. striata domestica	A0A218U883	KILTTALFSVLLLGTAL	
O. spaldingii	A0A7K8G0Q9	KILTTALFSVLLLGTAL	Birds
B. capensis	A0A7K9I2Y8	KILTTAVFSVLLLGTSL	Amphibians
X. laevis	A5PKQ2	KILTTALFSVLLLRKSL	Reptiles
P. guttatus	A0A6P9D2P8	KILTTAVFSVLMLRKSL	
S. salar	A0A1S3LGH5	KILTTALFSVLMLRKSL	
C. gobio	A0A6J2PFP1	KILTTALFSVMMLRKSL	
C. chanos	A0A6J2W8A0	KILTTALFSVMMLRKSL	
P. ranga	A0A6P7IV90	KILTTALFSVLMLRKSL	Ray-finned
C. magur	A0A8J4THQ4	KILTTALFSVLMLRKSL	fishes
I. punctatus	W5UFK5	KILTTALFSVLMLRKSL	
A. spatula	A0A8J7P5A1	KILTTAVFSVLMLQKSL	

Figure S2. Multiple sequence alignment (MSA) results - aligned human protein (P78381) with orthologues. Residues highlighted (Leucine with green, Valine with magenta) at position 154 in the human protein P78381.

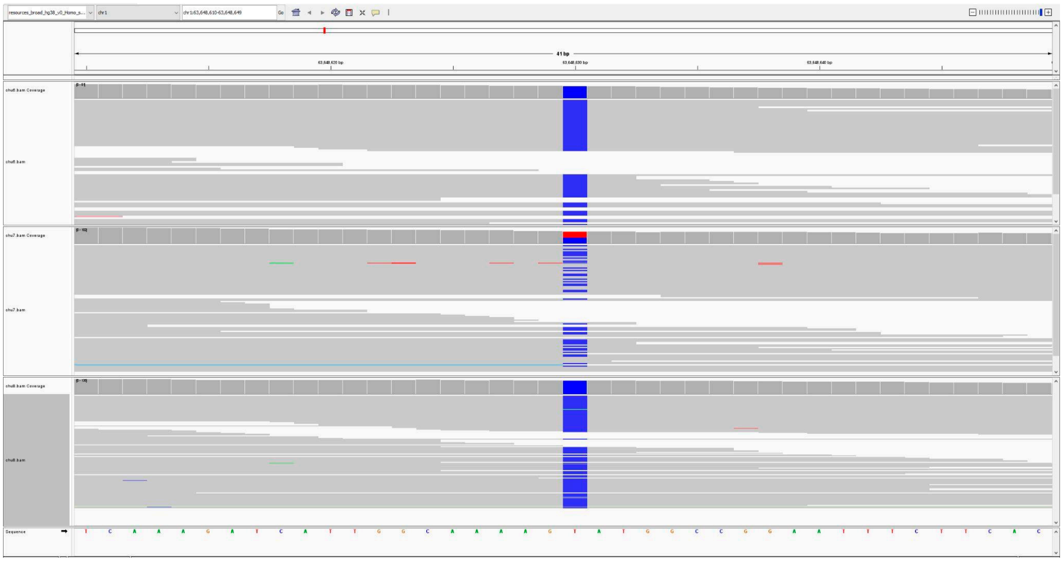

Figure S3. **Screenshot from Integrative genomics viewer [37] of the mutation at position chr1: 63648630 in gene *PGM1*.** The patient is labeled as chu6, parents are samples chu7 and chu8.

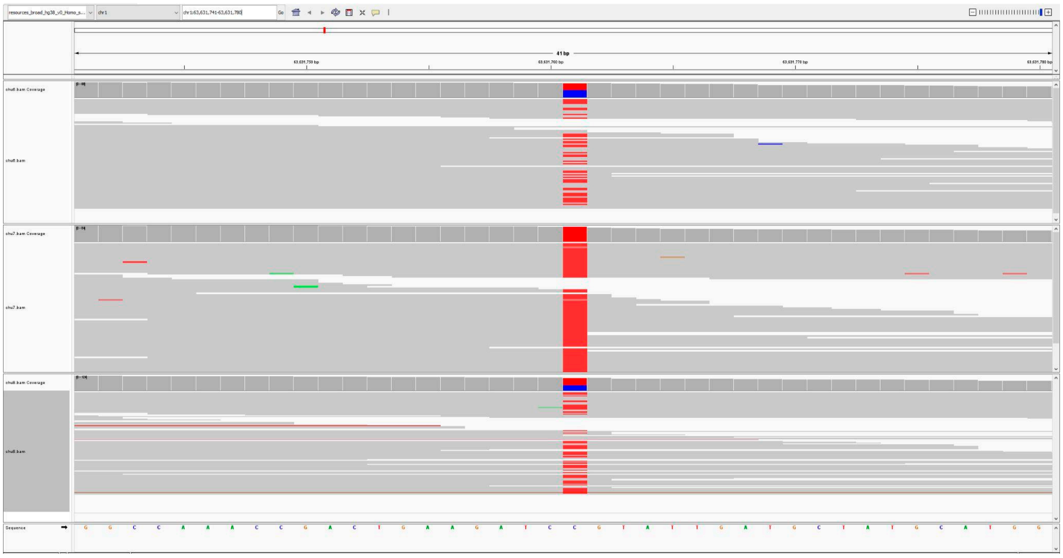

Figure S4. Screenshot from Integrative genomics viewer [37] of the mutation at position chr1: 63631761 in gene *PGM1*. The patient is labeled as chu6, parents are samples chu7 and chu8.

Subtype	SLC35A2-CDG presented patient	Galactosemia [32]	B4GALT1-CDG [33]	TMEM165-CDG [35, 36]	PGM1-CDG [36]	COGs-CDG [33, 36]
**Deficient enzyme**	UDP-galactose transporter	galactose-1-phosphate uridylyltransferase, galactokinase 1, UDP-galactose-4-epimerase	beta-1,4-galactosyltransferase 1	transmembrane protein 165	phosphoglucomutase 1	conserved oligomeric Golgi complex
**Increased serum N-glycan biomarker**						
Hex3HexNAc4 *m/z* 1661.9	+	+/-	+	+	+	-
Hex3HexNAc4Fuc1 *m/z* 1835.9	-	+/-	+	+	-	+
Hex3HexNAc5 *m/z* 1906.9	+	-	+	-	-	-
Hex3HexNAc5Fuc1 *m/z* 2081.1	+	-	+	-	-	-
Hex4HexNAc4 *m/z* 1865.1	+	+/-	-	+/-	-	+/-
Hex4HexNAc4NeuAc1 *m/z* 2227.2	+	-	+	+	+	+
Hex5HexNAc4NeuAc1 *m/z* 2431.2	-	-	-	+/-	+/-	+
Hex5HexNAc4NeuAc1 Fuc1 *m/z* 2605.3	-	-	-	+	-	+

Table S1. List of other diseases that could affect galactose metabolism and lead to similar serum glycoprofile as in SLC35A2-CDG, with respective increased levels (+) or normal levels (-) of N-glycan biomarkers. Fuc - fucose, Hex- hexose, HexNAc - N-acetylhexosamine, NeuAc - sialic acid.

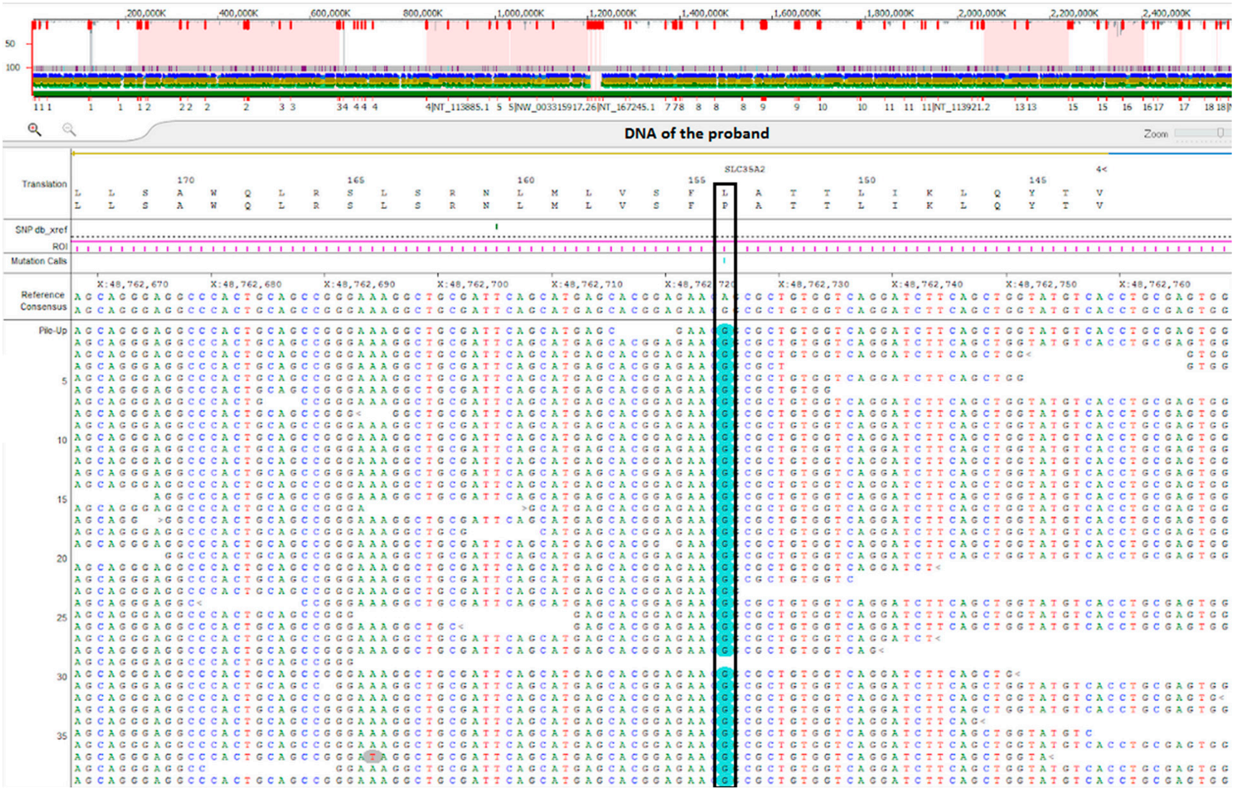

Figure S5. Analysis of DNA sequence of the selected region of SLC35A2 gene - proband.

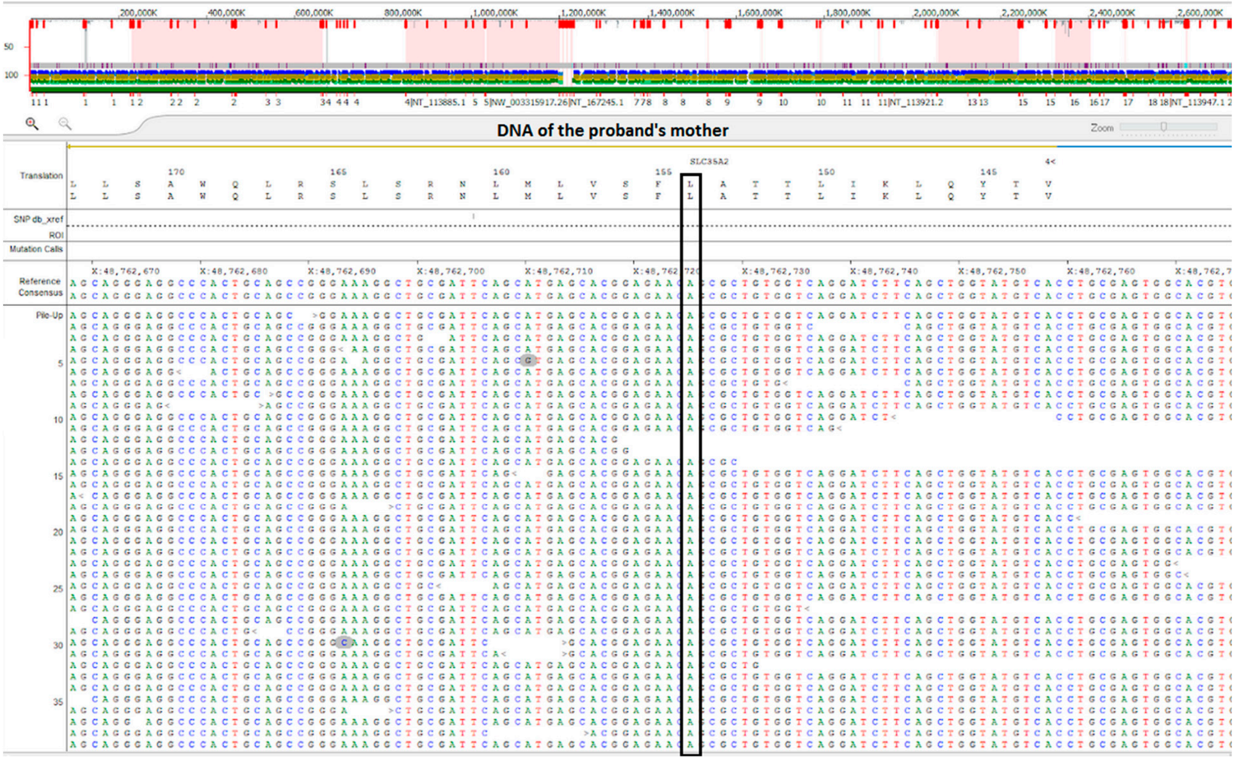

Figure S6. Analysis of DNA sequence of the selected region of SLC35A2 gene - mother.

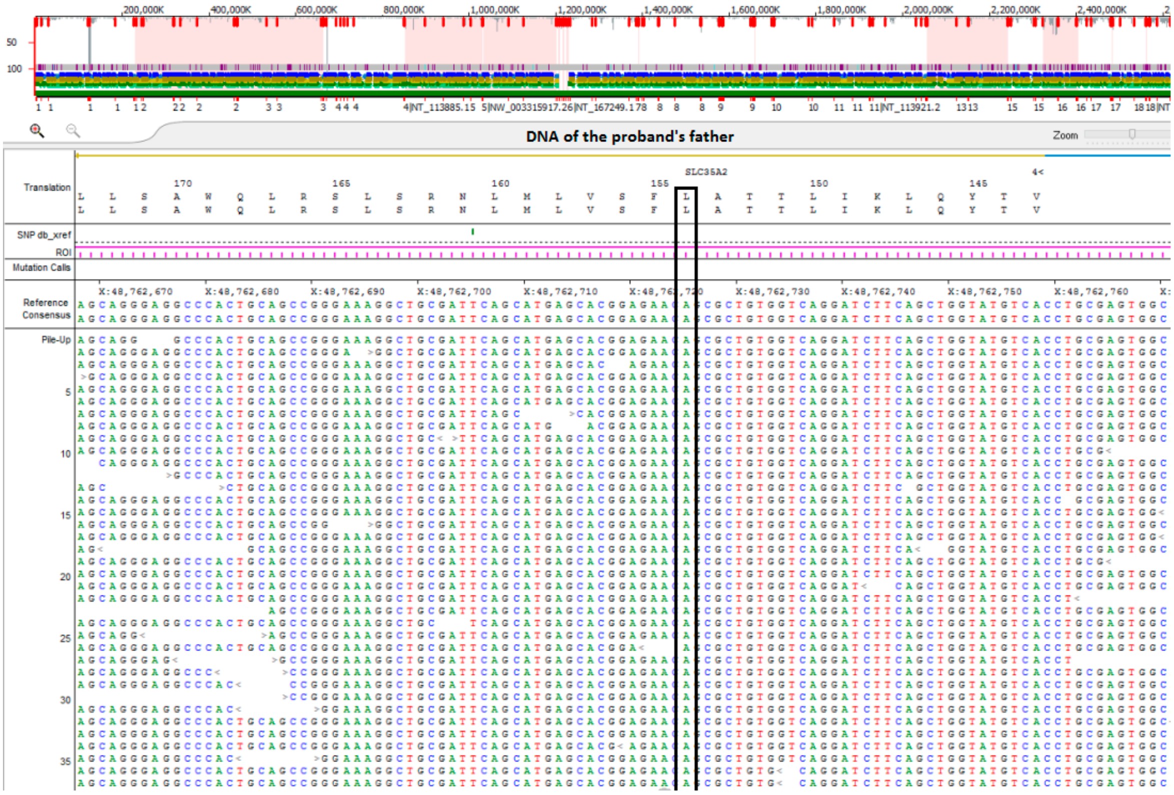

Figure S7. Analysis of DNA sequence of the selected region of SLC35A2 gene - father.
